# Risk factors for curable sexually transmitted infections among youth: findings from the STICH population survey in Zimbabwe

**DOI:** 10.1136/sextrans-2024-056146

**Published:** 2024-06-13

**Authors:** Kevin Martin, Ethel Dauya, Victoria Simms, Tsitsi Bandason, Steven Azizi, Anna Machiha, Tinei Shamu, Primrose Musiyandaka, Tinashe Mwaturura, Suzanna C Francis, Constance R S Mackworth-Young, Joanna Busza, Constancia Mavodza, Mandi Tembo, Richard J Hayes, Katharina Kranzer, Rashida A Ferrand, Chido Dziva Chikwari

**Affiliations:** 1 Clinical Research Department, The London School of Hygiene & Tropical Medicine, London, UK; 2 The Health Research Unit Zimbabwe, Biomedical Research and Training Institute, Harare, Zimbabwe; 3 MRC International Statistics and Epidemiology Group, Department of Infectious Disease Epidemiology, The London School of Hygiene & Tropical Medicine, London, UK; 4 AIDS and TB Unit, Zimbabwe Ministry of Health and Child Care, Harare, Zimbabwe; 5 Newlands Clinic, Harare, Zimbabwe; 6 Department of Global Health and Development, The London School of Hygiene & Tropical Medicine, London, UK; 7 Department of Public Health, Environments and Society, The London School of Hygiene & Tropical Medicine, London, UK; 8 Division of Infectious Diseases and Tropical Medicine, LMU University Hospital, LMU Munich, Munich, Germany

**Keywords:** AFRICA, Risk factors, Chlamydia Infections, Gonorrhea, TRICHOMONAS

## Abstract

**Abstract:**

**Objectives:**

Youth are at high risk of sexually transmitted infections (STIs) in Africa. We aimed to determine the risk factors for curable STIs in youth in Zimbabwe.

**Methods:**

A population-based survey was conducted among randomly selected 18–24 year-olds in 16 communities across two provinces in Zimbabwe to ascertain outcomes for a cluster randomised trial investigating the impact of community-based STI screening for youth on population prevalence of STIs. Participants underwent an interviewer-administered questionnaire, HIV testing and screening for *Chlamydia trachomatis* (CT), *Neisseria gonorrhoeae* (NG) and *Trichomonas vaginalis* (TV). Risk factors for curable STIs were explored through multivariable logistic regression.

**Results:**

Of the 5601 participants, 62.5% (n=3500) were female, and the median age was 20 (IQR 19–22) years. HIV prevalence was 6.3% (351/5556), and 55.4% (1939/3501) reported condomless sex at last intercourse. Only 7.2% (401/5599) reported STI symptoms, but CT/NG/TV prevalence was 19.8% (1107/5601). On multivariable analysis, factors associated with STI diagnosis included being aged 21–24 years (adjusted OR (aOR) 1.37, 95% CI 1.17 to 1.61); female sex (aOR 2.11, 95% CI 1.76 to 2.53); being unemployed/informally employed (compared with in education/formal employment) (aOR 1.35, 95% CI 1.13 to 1.61); increasing number of sexual partners in the preceding 12 months (one partner: aOR 2.23, 95% CI 1.73 to 2.88; two partners: aOR 2.39, 95% CI 1.69 to 3.39); living with HIV (aOR 1.44, 95% CI 1.07 to 1.94); and previous attempted suicide (aOR 1.58, 95% CI 1.08 to 2.32).

**Conclusions:**

The prevalence of STIs among youth in Zimbabwe is high, particularly among those with HIV. In addition to moving away from syndromic STI management and strengthening implementation of existing prevention tools, there is a need for a more holistic focus on broader risk factors such as mental health and employment opportunities, and of integration of HIV and STI programming.

**Trial registration number:**

ISRCTN15013425, NCT03719521.

**WHAT IS ALREADY KNOWN ON THIS TOPIC**

- The lack of availability of diagnostics in Southern Africa hampers surveillance of sexually transmitted infections (STIs) at a population level. In Zimbabwe, previous studies have shown high prevalence among youth attending sexual and reproductive health services, but it is unclear to what extent this is representative of the general youth population.

**WHAT THIS STUDY ADDS**

- This study confirms high population prevalence of curable STIs among youth in Zimbabwe. It demonstrates important risk factors for infection that are often not directly addressed such as lower education levels, lack of employment opportunities, living with HIV and mental health morbidity.

**HOW THIS STUDY MIGHT AFFECT RESEARCH, PRACTICE OR POLICY**

- These findings reinforce that current STI control strategies are failing youth. Alongside moving from syndromic to aetiological management of STIs, we must also ensure that STI and HIV services are more meaningfully integrated. As well as strengthening current control strategies, it is important to address broader factors such as mental health, education and employment opportunities for youth. This is also likely to have an impact on overall well-being.

## Background

 The incidence of curable sexually transmitted infections (STIs) remains high globally.^1 2^ This is in contrast to HIV, where technological advances, advocacy and sustained funding have contributed to a global decline in HIV incidence over the past two decades.^2^ Despite the commonalities between curable STIs and HIV, including mode of transmission, there has been much less support for STIs and programming has been heavily siloed.^2^

In Southern Africa, STI management still relies on a syndromic approach, which is the provision of treatment to an individual presenting with a collection of typical symptoms that may be caused by an STI, without establishing aetiology with a diagnostic test.^2 3^ However, as a result of the poor sensitivity and specificity of syndromic management, and its inability to detect asymptomatic infections, the majority of infections remain undiagnosed and untreated.^4 5^ The lack of aetiological data means that STI surveillance data on which to base policy are often lacking. Furthermore, aetiological data, if collected, are mainly from symptomatic individuals, and so are not representative of the general population.^6^ The combination of a lack of high-quality data, together with chronic underfunding of STI programmes, is a challenge to population-level STI control.

‘Youth’ is defined by the United Nations as those persons between the ages of 15 and 24 years, who are an important risk group for STIs.^7^ A 2018 meta-analysis among women participating in HIV prevention studies found that young women in East and Southern Africa were at higher risk of STIs than older age groups.^8^ We previously reported a prevalence of 16.5% of *Chlamydia trachomatis* (CT) and/or *Neisseria gonorrhoeae* (NG) among youth in Zimbabwe attending sexual and reproductive health (SRH) services where STI screening was offered.^4^ However, as screening was in individuals who sought care, their prevalence and risk factors may differ from the general population. Identification of risk factors at population level is important in planning STI control strategies and informing design of interventions such that individuals at highest risk can be targeted.

In this paper, we investigate risk factors for CT, NG and/or *Trichomonas vaginalis* (TV) infection among youth in Zimbabwe, within a population-based survey that tested young people for these STIs regardless of symptoms.

## Methods

### Study design and setting

As part of a cluster randomised trial (STICH (STIs in CHIEDZA) trial—registration number: ISRCTN15013425), a population-based survey was conducted to measure the impact of STI screening and treatment on population-level prevalence of any of CT, NG and TV (primary outcome) among youth aged 18–24 years in two provinces in Zimbabwe.^9^ The trial results, including prevalence of individual STIs, will be reported elsewhere.

The STICH trial was embedded within the CHIEDZA (Community based interventions to improve HIV outcomes in youth) cluster randomised trial (registration number: NCT03719521) which investigated the impact of providing a community-based package of integrated HIV and SRH services on population-level HIV outcomes. The CHIEDZA integrated service package included HIV testing and care and SRH services including family planning, condoms and risk reduction counselling.

The STICH trial was conducted in the Harare and Bulawayo provinces, with each province having eight clusters (defined as a geographically demarcated area in a community with a clinic and a community centre from where the intervention was delivered) randomised 1:1 to the intervention or standard of care. STI screening for CT (male and female), NG (male and female) and TV (female only) was offered to youth aged 16–24 years and resident in the intervention clusters over a 1-year period (21 September 2020 to 30 September 2021 in Harare, 4 January 2021 to 15 December 2021 in Bulawayo) as part of the CHIEDZA integrated service package. The service was delivered from community centres and STI screening was offered regardless of whether attendees had symptoms. Syndromic management was also offered to those who presented with symptoms following national guidelines.^3^ Treatment and partner notification were provided free of charge to those diagnosed with an STI.^9^ STI management in the standard of care arm consisted of syndromic management at public sector facilities.

### Prevalence survey study procedures

The postintervention prevalence survey was conducted over 3 months within each province between October 2021 and March 2022. The survey age criteria were chosen to ensure maximum exposure to the intervention.^9^ Research teams first visited communities to inform residents about the survey. Subsequently, all households (defined as a person or group of related or unrelated persons living together in the same dwelling or unit(s) of a dwelling, who acknowledge one adult male or female as head of the household, who share the same housekeeping arrangements and who are considered a single unit) in randomly selected street segments in a cluster were enumerated. All individuals aged 18–24 years residing in the enumerated households were eligible to participate. If a potentially eligible individual was unavailable, up to three repeat visits were made to enrol the individual.

Data on sociodemographic and clinical factors, as well as sexual behaviour, were collected using an interviewer-administered questionnaire, using SurveyCTO on tablets. The survey was piloted in youth prior to use in the prevalence survey. The relevant survey questions and response categories for this study are shown in online supplemental table A.

Sample size was powered for the STICH trial outcome to provide 80% power to detect a reduction in the population-level prevalence of CT/NG/TV from 17% to 10% between control and intervention arms.^9^ The STICH survey was nested within the larger CHIEDZA survey (powered to detect an increase in prevalence of HIV viral suppression), which aimed to recruit 700 participants per cluster. For STICH, we aimed to recruit a total of 300 participants per cluster. Therefore, on randomly selected survey days, participants were asked to provide a urine sample for STI screening. CT, NG and TV screening was performed using the multiplex Seegene assay (Seegene, South Korea). A dried blood spot was collected and stored for later HIV antibody testing, and all individuals who did not know their HIV status received pretest counselling and were referred to their nearest health facility for HIV testing.

### Statistical analysis

STATA V.18.0 (StataCorp, Texas, USA) was used for data analysis. An individual was defined as having any STI (primary outcome) if they tested positive for at least one STI (CT, NG or TV). If a test result for one or more STIs was not available, a participant was excluded from analyses, unless one of the remaining tests was positive. STI prevalence, weighted by province, age and sex, was also calculated using all 6817 enumerated individuals as the target.

**Figure 1:**
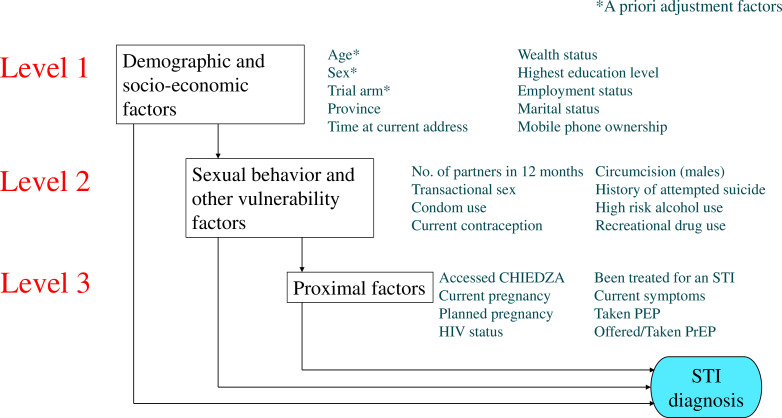
Conceptual hierarchical framework of factors associated with sexually transmitted infection (STI) test positivity among young people in Zimbabwe. CHIEDZA, community based interventions to improve HIV outcomes in youth: a cluster randomised trial in Zimbabwe; PEP, postexposure prophylaxis for HIV; PrEP, pre-exposure prophylaxis for HIV; STI, sexually transmitted infection.

Univariable and multivariable logistic regressions were used to investigate factors associated with a positive STI result. A three-level hierarchical conceptual approach was used to structure the analysis (figure 1).^4^ A household wealth indicator, based on the presence of eight household assets, was developed using principal components analysis.

Age, sex and trial arm were considered a priori risk factors. Level 1 (distal) factors included demographic and socioeconomic factors, including province, employment status, marital status, education level and economic status, due to previously established associations with STI risk in other populations.^10^,^14^ ‘Time lived at current address’ was also included to account for potential vulnerabilities created by migration and mobility.^15^

Level 2 factors related to sexual behaviours and related factors that were either risky or protective, including condom use, number of sexual partners, transactional sex and male circumcision.^10 11 16^ Alcohol and substance use and depression may also affect risk behaviours and therefore affect STI risk.^11 17 18^

Level 3 (proximal) factors were individual attributes that themselves were likely affected by an individual’s sexual and other risk behaviours, including pregnancy, HIV status, current STI symptoms or engagement with services.

For level 1, all age, sex and trial arm-adjusted sociodemographic factors that met a p value cut-off of <0.1 were included in the multivariable model for level 1. After adjusting for these factors, the variables that were associated at <0.1 became the ‘core’ level 1 factors. All level 1-adjusted level 2 (behavioural) factors that met a p value cut-off of <0.10 were included in the multivariable model for level 2, together with the core level 1 factors. Associations with level 3 factors were determined in a similar way.

If factors restricted by prior sexual intercourse, or by sex, met the p value cut-off and that factor was needed to adjust other variables, a reference category was added to the variable so that there would be an unrestricted adjusted model. For example, for male circumcision, a ‘female’ reference category was added to the variable when included in the adjusted models. Missing data were otherwise excluded pairwise. All regression analyses were adjusted for clustering at the street segment level.

Additional multivariate analyses were conducted in male and female participants separately to investigate sex-specific factors associated with a positive STI result. Post hoc analyses were also performed to explore the effect of sociodemographic and economic factors on CHIEDZA attendance within the trial arm, to compare individuals who attended CHIEDZA to those who did not.

## Results

### Participant characteristics

**Figure 2:**
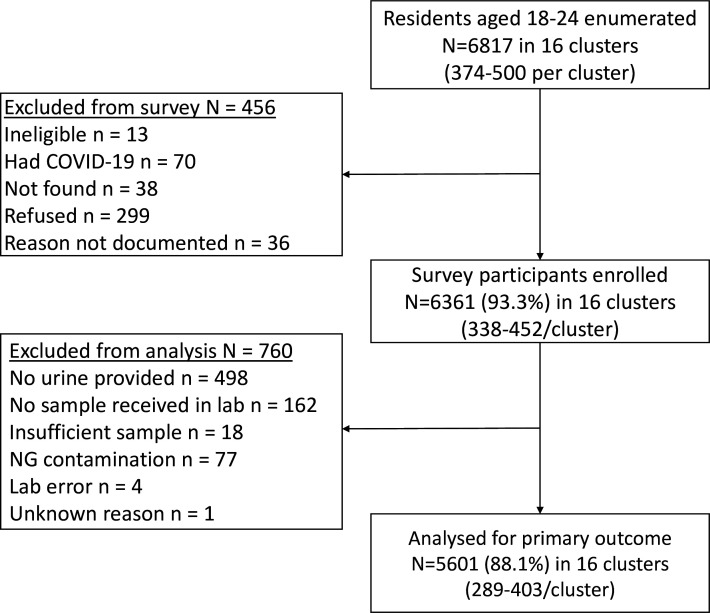
Study Consolidated Standards of Reporting Trials (CONSORT) diagram. NG, *Neisseria gonorrhoeae.*

The Consolidated Standards of Reporting Trials diagram is shown in figure 2. Of the 6817 enumerated individuals, 6361 (93.3%) were enrolled, of whom 5601 (88.1%) were assessed for the primary outcome. Of these, 2710 (48.4%) were enrolled from the Harare province, and 2756 (49.2%) were in the intervention arm. Among those in the intervention arm, 27.2% (749/2756) stated that they had accessed CHIEDZA services, compared with 0.6% (17/2845) in the control arm. The HIV prevalence was 6.3% (351/5556).

The characteristics and sexual risk factors of participants are shown in table 1. The majority (62.5%; 3500/5601) were female, and the median age was 20 (IQR 19–22) years. One-third (1801/5601) of participants reported having lived at their current address for less than 2 years. Over half (51.3%; 2874/5601) of participants reported not being in education or having any formal or informal employment, and 49.7% (2415/4855) had a household income of less than US$100 per month.

**Table 1:** Characteristics of participants recruited into the study (n=5601 unless otherwise stated)

Variable	n (%)
**Demographic and socioeconomic factors**	
Sex	
Female	3500 (62.5)
Male	2101 (37.5)
Age (years)	
18–20	2855 (51.5)
21–24	2716 (48.5)
Time lived at current address (months)	
<12	1299 (23.2)
12–24	502 (9.0)
>24	3800 (67.8)
Wealth quintile (n=5592)	
1 (poorest)	903 (16.1)
2	1112 (19.9)
3	1211 (21.7)
4	1220 (21.8)
5 (richest)	1146 (20.5)
Highest completed education level	
Up to primary	1061 (18.9)
Secondary	4120 (73.6)
Postsecondary	420 (7.5)
Current employment status	
In education	1526 (27.2)
Formal employment	245 (4.4)
Informal employment	956 (17.1)
None of the above	2874 (51.3)
Marital status	
Never married	4280 (76.4)
Married or living together as if married	1084 (19.4)
Divorced, widowed or separated (and currently unmarried)	237 (4.2)
Mobile phone ownership (n=5596)	
Owns mobile phone	4968 (88.8)
Shares mobile phone with someone else	342 (6.1)
Does not use a mobile phone	286 (5.1)
**Sexual behaviour, vulnerabilityand proximal factors**	
Ever had sexual penetrative intercourse (n=5573)	
Yes	3816 (68.5)
Number of sexual partners in the last 12 months (n=5530)	
0	2016 (36.5)
1	2580 (46.6)
2	532 (9.6)
≥3	402 (7.3)
Received or provided gifts, financial support or other material support in exchange for sex in the last 12 months (n=5262)	
Yes	117 (2.2)
Condom use in the last 12 months during vaginal or anal sex (n=3505)	
Most of the time	1357 (38.7)
Sometimes (about half the time)	699 (19.9)
Rarely or never	1449 (41.3)
Condom use at last sexual intercourse (n=3501)	
Yes	1562 (44.6)
Females only: planning of most recent pregnancy (n=1539)	
Wanted to become pregnant	803 (52.2)
Would have preferred to put it off for a while	288 (18.7)
Did not want to become pregnant	448 (29.1)
Males only: been circumcised (n=2076)	
Yes	1142 (55.0)
History of attempted suicide (n=5587)	
Yes	169 (3.0)
High-risk alcohol consumption* over the last 12 months (n=5593)	
Yes	178 (3.2)
Frequency of recreational drug use (n=5592)	
Daily	172 (3.1)
Several times a week	121 (2.2)
Monthly to every few months	114 (2.0)
Once or twice a year	68 (1.2)
Never	5117 (91.5)
Ever been treated for a sexually transmitted infection (n=3807)	
Yes, in the last year	223 (5.9)
Yes, more than a year ago	114 (3.0)
No	3470 (91.1)
Current symptoms (at least one of dysuria, vaginal/urethral discharge, abnormal vaginal smell, genital itch, genital sore/blister/bump/wart/growth) (n=5592)	
Yes	401 (7.2)
Ever been offered PrEP (n=5584)	
Yes	116 (2.1)

* High-risk alcohol consumption defined as: drinking 4 or more times per week, having 7 or more drinks on a typical day when drinking, or having 6 or more drinks daily or almost daily.

PrEPpre-exposure prophylaxis

Two-thirds (68.5%; 3816/5573) reported ever having had penetrative sexual intercourse, of whom 23.7% (906/3816) tested positive for an STI. Of those not reporting having had penetrative intercourse, 11.2% (197/1757) had an STI. Of those reporting having had sex, over half (55.3%; 1939/3505) reported no condom use at last sexual intercourse. Under half of all women had had at least one pregnancy (44.2%; 1544/3497) and a fifth (20.7%; 724/3500) were currently using contraceptives (excluding condoms). Transactional sex was reported by 2.2% (117/5145) of participants.

### Risk factors associated with diagnosis of an STI

The prevalence of at least one of CT, NG or TV was 19.8% (95% CI 18.7% to 20.8%; 1107/5601). Prevalence weighted by province, age and sex was 19.7% (95% CI 18.7% to 20.7%). Univariable associations with STI diagnosis are shown in online supplemental tables B–D.

The final multivariable model is shown in table 2. Characteristics associated with an increased prevalence of STIs in the final model include: being aged 21–24 (adjusted OR (aOR) 1.37, 95% CI 1.17 to 1.61); female sex (aOR 2.11, 95% CI 1.76 to 2.53); living in Bulawayo (aOR 1.23, 95% CI 1.05 to 1.44); being unemployed or in informal employment (compared with being in education or formal employment) (aOR 1.35, 95% CI 1.13 to 1.61); having one (aOR 2.23, 95% CI 1.73 to 2.88), two (aOR 2.39, 95% CI 1.69 to 3.39) or three or more (aOR 3.05, 95% CI 2.09 to 4.44) sexual partners compared with none in the last 12 months; previous attempted suicide (aOR 1.58, 95% CI 1.08 to 2.32); living with HIV (aOR 1.44, 95% CI 1.07 to 1.94); current symptoms of an STI (aOR 1.43, 95% CI 1.11 to 1.84); and having been previously offered pre-exposure prophylaxis (PrEP) (aOR 1.61, 95% CI 1.06 to 2.43). There was also evidence that male circumcision (OR 0.63, 95% CI 0.45 to 0.88) and having a secondary (aOR 0.79, 95% CI 0.65 to 0.95) or postsecondary (aOR 0.77, 95% CI 0.54 to 1.09) education, compared with primary or less, were protective factors.

**Table 2:** Age, sex and trial arm-adjusted and multivariable associations between variables and chlamydia, gonorrhoea and/or trichomoniasis infection among youth in Zimbabwe (n=5164 in the final model)

Variable	STI prevalencen (%)	Age/sex/trial arm-adjusted OR (95% CI)	P value	Final adjusted OR (95% CI)	P value
**A priori factors**					
Age (years)			<0.001		0.0010
18–20	498/2885 (17.3)	1.00		1.00	
21–24	609/2716 (22.4)	1.40 (1.21 to 1.63)		1.37 (1.17 to 1.61)	
Sex			<0.001		<0.001
Male	260/2101 (12.4)	1.00		1.00	
Female	847/3500 (24.2)	2.22 (1.87 to 2.65)		2.11 (1.76 to 2.53)	
Trial arm			0.16		0.21
Control	569/2845 (20.0)	1.00		1.00	
Intervention	538/2756 (19.5)	0.89 (0.76 to 1.05)		0.90 (0.77 to 1.06)	
Sociodemographic and economic factors (level 1)*					
Province			0.046		0.012
Harare	524/2710 (19.3)	1.00		1.00	
Bulawayo	583/2891 (20.2)	1.17 (1.00 to 1.36)		1.23 (1.05 to 1.44)	
Time lived at current address (years)			0.028		0.073
>2	686/3800 (18.1)	1.00		1.00	
<2	421/1801 (23.4)	1.19 (1.02 to 1.40)		1.16 (0.99 to 1.37)	
Highest completed education level			0.0011		0.042
Completed primary or less	262/1061 (24.7)	1.00		1.00	
Secondary	775/4120 (18.8)	0.74 (0.61 to 0.88)		0.79 (0.65 to 0.95)	
Postsecondary	70/420 (16.7)	0.62 (0.45 to 0.85)		0.77 (0.54 to 1.09)	
Current employment status			<0.001		0.0010
In education or formal employment	267/1771 (15.1)	1.00		1.00	
Informal or no employment	840/3830 (21.9)	1.42 (1.21 to 1.67)		1.35 (1.13 to 1.61)	
Marital status			0.039		0.081
Never married	765/4280 (17.9)	1.00		1.00	
Married or living together as if married	268/1084 (24.7)	1.04 (0.87 to 1.25)		0.94 (0.77 to 1.13)	
Divorced, widowed or separated (and currently unmarried)	74/237 (31.2)	1.50 (1.10 to 2.04)		1.37 (0.98 to 1.90)	
**Sexual behaviour and other vulnerability factors (level 2)†**					
Number of sexual partners in the last 12 months (n=5530)			<0.001		<0.001
0	238/2016 (11.8)	1.00		1.00	
1	647/2580 (25.1)	2.32 (1.90 to 2.82)		2.23 (1.73 to 2.88)	
2	114/532 (21.4)	2.57 (1.87 to 3.52)		2.39 (1.69 to 3.39)	
≥3	91/402 (22.6)	3.36 (2.40 to 4.70)		3.05 (2.09 to 4.44)	
Condom use in the last 12 months during vaginal or anal sex (n=3505)			0.047		0.044
Most of the time	284/1357 (20.9)	1.00		1.00	
Sometimes (about half the time)	189/699 (27.0)	1.34 (1.05 to 1.71)		1.34 (1.07 to 1.72)	
Rarely or never	375/1449 (25.9)	1.03 (0.83 to 1.27)		1.22 (0.96 to 1.55)	
Males only: been circumcised (n=2076)			0.096		0.0068
No	129/934 (13.8)	1.00		1.00	
Yes	128/1142 (11.2)	0.78 (0.58 to 1.05)		0.63 (0.45 to 0.88)	
History of attempted suicide (n=5587)			<0.001		0.019
No	1047/5418 (19.3)	1.00		1.00	
Yes	56/169 (33.1)	1.89 (1.33 to 2.70)		1.58 (1.08 to 2.32)	
**Proximal factors (level 3)** ‡					
Accessed CHIEDZA health services			0.13		0.049
Yes	136/766 (17.8)	1.00		1.00	
No	971/4835 (20.1)	1.20 (0.95 to 1.53)		1.29 (1.00 to 1.65)	
Females only: most recent pregnancy planning (n=1534)			0.0064		0.11
Planned pregnancy	210/803 (26.2)	1.00		1.00	
Unplanned or would have preferred to wait	235/736 (31.9)	1.41 (1.10 to 1.81)		1.24 (0.95 to 1.62)	
HIV status (n=5556)			<0.001		0.018
Negative	987/5205 (19.0)	1.00		1.00	
Positive	111/351 (31.6)	1.68 (1.28 to 2.22)		1.44 (1.07 to 1.94)	
Presence of any current symptoms (n=5592)			<0.001		0.0057
No	982/5191 (18.9)	1.00		1.00	
Yes	124/401 (30.9)	1.70 (1.33 to 2.16)		1.43 (1.11 to 1.84)	
Been offered PrEP (n=5585)			<0.001		0.025
No	1066/5468 (19.5)	1.00		1.00	
Yes	40/116 (34.5)	1.99 (1.33 to 2.96)		1.61 (1.06 to 2.43)	

* Sociodemographic and economic factors (level 1) are only adjusted for a priori factors (age group, sex and trial arm) and other level 1 factors (province, time lived at current address, education level, employment status and marital status).

† Sexual behavioural and other vulnerability factors (level 2) are adjusted for the level 1 factorsand alsolevel 2 factors (history of attempted suicide, number of sexual partners in preceding 12 months, condom use, and male circumcision.).

‡ Proximal factors (level 3) are adjusted for the listed level 1 and level 2 factors, as well as other level 3 factors (if accessed CHIEDZA services, pregnancy planning, HIV status, current symptoms, and if have been offered HIV pre-exposure prophylaxis.PrEP).

PrEPpre-exposure prophylaxisSTIsexually transmitted infection

Separate models for female and male participants are shown in online supplemental tables E and F, respectively. In both final models, age, number of sexual partners in the past 12 months, history of attempted suicide and presence of current symptoms were associated with STI diagnosis. In the male model only, province (aOR 1.43, 95% CI 1.06 to 1.93) and circumcision status (male only variable) (aOR 0.65, 95% CI 0.47 to 0.90) were associated with STI diagnosis. In the female model only, employment status (aOR 1.60, 95% CI 1.30 to 1.95), using condoms sometimes (aOR 1.44, 95% CI 1.08 to 1.93) compared with most of the time, not accessing CHIEDZA (aOR 1.34, 95% CI 1.02 to 1.75), HIV status (aOR 1.44, 95% CI 1.03 to 2.01) and if offered PrEP (aOR 1.72, 95% CI 1.08 to 2.74) were associated with STI diagnosis.

### Post hoc analyses

Univariable associations between sociodemographic and economic factors and attendance at CHIEDZA services within the trial intervention arm are shown in online supplemental table G. Factors associated with attendance included female sex (OR 1.52, 95% CI 1.22 to 1.90) and having a secondary education (OR 1.42, 95% CI 1.11 to 1.81) compared with primary education only. Having lived at the current address for less than 2 years was associated with non-attendance (OR 0.53, 95% CI 0.42 to 0.66).

## Discussion

Nearly one in five young people were found to have at least one curable STI in this population-based survey in Zimbabwe. Important risk factors in the final model included sociodemographic and economic factors, including educational attainment, employment status and marital status. Campaigns and programmes to reduce both STIs and HIV often focus on sexual behaviours such as promotion of condom use and reduction in partner numbers. However, this approach risks neglecting important social and structural factors that may have a significant impact on STI risk. For example, economic disenfranchisement or lack of educational opportunities may lead to young women becoming engaged in relationships with an embedded power imbalance, where condom negotiation may be difficult or impossible.^19^ Addressing these upstream factors, such as by providing enhanced education and employment opportunities, may therefore lead to a reduction in STI risk, alongside broader benefits to both individuals and their communities. Given the varying risk factor profiles by sex, consideration should also be given to how intervention packages are differentiated for male and female youth to ensure resources are targeted effectively.

In multivariable analyses, risk factors for an STI included being in an older age group, being female, having increasing numbers of sexual partners and being uncircumcised (for males). These findings are congruent with the broader literature. Female sex is associated with STIs across many settings, and number of partners is a well-recognised risk factor for STIs.^1 11^ Additionally, among youth in Southern Africa, being in an older age group has been shown to be associated with STI diagnosis.^10 12^ Male circumcision has also been reported to be protective against STIs.^16^

We found that female youth who had been offered PrEP were more likely to have an STI, suggesting that where PrEP is available, it is being offered to those at higher risk,^20^ although it is important to note that the number of individuals who had been offered PrEP was very low. Low levels of condom and PrEP use suggest that young people in Zimbabwe are at ongoing high risk of both STIs and HIV. Additionally, the high proportion of unplanned pregnancies (47.8%) in conjunction with low levels of contraceptive use suggests this population has a high degree of unmet contraceptive need.

Having previously attempted suicide was associated with having an STI. This is consistent with the literature and demonstrates the interconnectedness of sexual and mental health.^11 21^ Huang *et al* previously showed in a large cohort study in Taiwan that there was a higher incidence of STI acquisition among individuals with depression than those without. Depression and other mental illness may influence risk perception, self-esteem and ability to both initiate and negotiate conversations surrounding safer sex behaviour, such as condom use, with moderate/severe depression symptoms shown to be associated with risk behaviours including condomless sex.^21 22^ Attempted suicide is a marker of severe depression, so these participants may have been particularly vulnerable and at risk of exploitation. Conversely, being in an exploitative or abusive relationship may increase the risk of both STI acquisition and suicide risk.^23 24^ This emphasises the need to develop holistic services that address the multifactorial needs of young people, rather than vertical programmes.

We had anticipated that STI prevalence might be lower at a population level compared with those attending CHIEDZA services due to healthcare-seeking behaviour of those more at risk. In keeping with this, overall population prevalence among women in this survey (24.2%) was slightly lower than age-matched young women attending CHIEDZA services (25.4%).^25^ However, within this survey, we found higher prevalence of STIs among individuals who did not attend CHIEDZA (20.1%) compared with those who did (17.8%). This could be due to those engaging with services also being more aware of, and engaging in, safer sexual behaviours. It could also indicate that young people most at risk of STIs face additional barriers to access, even to a free community-based service such as CHIEDZA. Our post hoc analyses (online supplemental table G) somewhat support this, with individuals with a secondary education more likely to have attended than those with primary education only. However, no association was found between attendance and other economic factors.

The association between HIV status and curable STIs is unsurprising given that alongside shared transmission routes, STIs also facilitate HIV transmission.^26^ Importantly, as simpler diagnostics make their way into the market, aetiological approaches to STI management will be increasingly implemented in resource-constrained settings. However, given the resource commitments required to implement an aetiological approach, it is likely that STI screening will, at least initially, focus on key populations at particular risk of STIs, before wider scale implementation. HIV clinics could potentially be an initial target group for implementation, providing lessons for other clinic-based settings. STI control is also an integral part of any HIV control strategy, and it is essential that integrated control strategies are developed. The high prevalence highlights that current STI control strategies are insufficient for population-level STI control. High STI prevalence has also been reported in young people and pregnant women across Southern Africa.^4 12 27^ This suggests a broader regional epidemic that may benefit from a coordinated multinational response and advocacy for STI control.

The study had several strengths. This was a large population-based survey with a high participation rate and a large sample size. STI screening was performed regardless of symptoms in all participants, enabling true STI prevalence estimates among youth. We acknowledge several limitations. There is evidence of social desirability bias, with over 1 in 10 (11.2%) participants who reported never having had penetrative sex having an STI. Similarly, transactional sex, despite using a broad definition including both money and gifts, may have been under-reported.^28^ History of attempted suicide was also much lower than other regional estimates.^29^ The questionnaire had significant input from young people and was piloted, but other approaches such as Audio Computer-Assisted Self-Interview may facilitate more accurate responses to sensitive questions. Furthermore, testing for CT/NG/TV using urine samples has lower sensitivity compared with vaginal swabs, so prevalence may have been underestimated.^30^ Generalisability of the results may also have been influenced by this being a postintervention survey to ascertain trial outcomes. Although we controlled for trial arm a priori in analyses, the intervention itself may have had complex downstream effects affecting STI prevalence and associated factors that are difficult to control for.

We have demonstrated a high prevalence of STIs at population level among youth in Zimbabwe. Addressing this will require a multipronged approach. Realistically, STI control will require significant investment and structural change, both in terms of healthcare delivery, but also a holistic approach that addresses the multiple needs of young people, particularly education, employment and social and mental health. Consideration must also be given to how best to target STI screening prior to a wider scale roll-out, given that STIs are distributed throughout the population, including in individuals without obvious risk factors.

## supplementary material

10.1136/sextrans-2024-056146 online supplemental file 1

## Data Availability

Data are available upon reasonable request.
