# Supplementary material for: Risk factors for curable sexually transmitted infections among youth: findings from the STICH population survey in Zimbabwe
Source: Sex Transm Infect. 2024 Jun 13;100(8):e056146. doi: 10.1136/sextrans-2024-056146 (PMC11671898; doi:10.1136/sextrans-2024-056146)
Supplement: online supplemental file 1 [file sextrans-100-8-s001.pdf]

Table of Contents

Supplementary table A: Relevant questions from the endline survey ... 2

Supplementary table B: Univariable and multivariable associations between sociodemographic and economic factors and presence of an STI, among young people in Zimbabwe ... 8

Supplementary table C: Univariable and multivariable associations between sexual behaviour and other vulnerability factors and presence of an STI, among young people in Zimbabwe ... 10

Supplementary table D: Univariable and multivariable associations between proximal factors and presence of an STI, among young people in Zimbabwe ... 12

Supplementary table E: Univariable, age- and trial arm-adjusted, and multivariable associations between variables and presence of an STI, among female youth in Zimbabwe ... 14

Supplementary table F: Univariable, age- and trial arm-adjusted, and multivariable associations between variables and presence of an STI, among male youth in Zimbabwe ... 17

Supplementary table G: Univariable associations between sociodemographic and economic factors and whether an individual has accessed CHIEDZA services within the trial arm (N = 2756 unless otherwise stated) ... 19

Supplementary table A: Relevant questions from the endline survey

**Question**	**Response categories**
**Sociodemographic**	
**How old are you?**<br>*Calculate age in completed years – must be between 18-24years*	
**How do you identify yourself? (select one)**	- Male<br>- Female<br>- Non-binary<br>- Transgender
**What is the sex you were assigned to at birth i.e. what is your biological sex? (select one)**	- Male<br>- Female<br>- Intersex
**How long have you lived in this current address? (select one)**	- Less than 12 months<br>- 12 months to 24 months (2 years)<br>- >2 years to 3 years<br>- More than 3 years
**Thinking about where you lived before you moved to this address, which one applies (select one):**	- I have always only lived at this address<br>- I lived in the same suburb, but different address<br>- I lived in the same town or city but not in the same suburb<br>- I lived outside this town/city
**What would you say your average regular household income (in United States Dollar equivalent) would be, per month? (select one)**<br>*A household defined as person or group of related or unrelated persons who live together in the same dwelling or unit(s) of a dwelling, who acknowledged one adult male or female as head of the household, who share the same housekeeping arrangements, and who are considered a single unit*	- Less than USD 50<br>- USD50-100<br>- USD101-200<br>- USD 201-500<br>- USD 501-900<br>- More than USD 900<br>- Don't know/Don't want to say
**Does your household have the following working items? (Yes/No for each option)**	- Fridge (Yes/No)<br>- Bicycle (Yes/No)<br>- Car/truck (Yes/No)<br>- Television (Yes/No)<br>- Radio (Yes/No)<br>- Microwave (Yes/No)<br>- Cell phone (Yes/No)<br>- Computer or laptop or tablet (Yes/No)
**What is the highest level of education you have completed? (select one)**<br>*If still in education, tick the highest level completed: e.g. if currently in Secondary Form 6, then the highest completed is Secondary Form 5*	- Never attended school<br>- Primary Grade 1<br>- Primary Grade 2<br>- Primary Grade 3<br>- Primary Grade 4

	- Primary Grade 5<br>- Primary Grade 6<br>- Primary Grade 7<br>- Secondary Form 1<br>- Secondary Form 2<br>- Secondary Form 3<br>- Secondary Form 4<br>- Secondary Form 5<br>- Secondary Form 6<br>- Vocational / trade school<br>- College (diploma, certificate)<br>- University
**Currently, what is the main activity you are engaged in? (select one)**<br>*Employed defined as having own registered business or work in the past seven days that generates a regular income. Includes not having worked in the past seven days but are regularly employed but were absent from work for leave, illness, vacation, or other such reason. Informal sector defined as not having a "regular" income.*	- In education (university/college/school)<br>- Owns a registered business or has a formal job that pays a wage i.e. employed<br>- Work in informal sector (e.g. subsistence farming, informal income-generating activities)<br>- None of the above
**What is your marital status? (select one)**	- Married or living together as if married<br>- Never married<br>- Divorced, widowed or separated (and currently unmarried)
**CHIEDZA knowledge and access**	
**Have you ever heard of CHIEDZA?**<br>*CHIEDZA refers to an initiative/programme that provides weekly health services for young people in the community, not in clinics. If the response is No, probe by showing the CHIEDZA UKUKHANYA logo and mentioning the name of the community centre from where CHIEDZA is delivered in the cluster and the day of the week CHIEDZA operated in that cluster. If interviewing in a control cluster, mention the names of the 4 intervention centres in the province you are interviewing in.*	- Yes<br>- No
**Have you ever accessed CHIEDZA health services delivered from community centres/halls? *Access is defined as having attended CHIEDZA, even if no services were taken up***<br>*Participants may not know the name of the services but may have accessed it- probe as follows: CHIEDZA provides HIV testing, condoms, STI testing and Treatment, menstrual products, contraception, health counselling in community centres/halls. Fingerprints are taken before services are taken before you get a service in the same way you had a fingerprint taken by me.*	- Yes<br>- No<br>- Not applicable, have not heard about CHIEDZA
**Pregnancy and contraception (females only)**	
**Are you pregnant at the moment?**	- Yes<br>- No
**Have you ever been pregnant (excluding current pregnancy)?**<br>*Include pregnancies that did not go to full term (i.e. miscarriage, termination of pregnancy/result in birth?*	- Yes<br>- No
**Thinking about your most recent pregnancy (or this pregnancy if you are currently pregnant if applicable), which sentence best describes your situation at that time?**	- I wanted to become pregnant<br>- I would have preferred to put it off for a while<br>- I did not want to become pregnant

	- Don’t know /Don’t want to say
**What contraceptive method(s) are you (or your partner) currently using?**<br>*Read through all options and Tick all that apply*	- Male condom<br>- Female condom<br>- Oral contraceptive pills (daily)<br>- Injection (Depo)<br>- Implants (Jadelle/Norplant)<br>- IUD/Loop<br>- Diaphragm or ring<br>- Foam/jelly<br>- Male sterilisation<br>- Female sterilisation<br>- Breastfeeding / Lactational amenorrhoea method<br>- Rhythm method/Safe days (avoiding sex on days I am fertile)<br>- Withdrawal method (partner withdraws before ejaculation)<br>- Other Traditional method [Specify]<br>- Other modern method [Specify]<br>- Don’t want to say<br>- Not using contraception to prevent pregnancy<br>- Not applicable, am not sexually active
**Sexual health and HIV prevention**	
**How old were you when you first had penetrative sexual intercourse, if ever?**<br>***By this I mean when a man/husband/boyfriend/partner put his penis inside a woman’s vagina (vaginal sex), or inside a woman or a man’s anus or backside (anal sex). Sex can be between two people of the same gender and can have occurred even if someone was not sure they wanted it.***<br>*If reports NEVER, probe: At what age did someone first have sexual intercourse with you, if ever?*	- Record age _____<br>- I can’t remember the age when I first had penetrative sex<br>- Don’t want to say<br>- I have not ever had penetrative sex
**In your lifetime, how many different sexual partners have you had (including regular partner if applicable)?**<br>*Sexual partner refers to someone whom you have vaginal or anal sex with*	
**In the past 12 months, with how many people have you had sex?**	
**People enter into sexual relations / have sex (vaginal or anal sex) for many different reasons. In the last 12 months, have you had sex or been sexually involved with anyone because he or she gave you or told you he or she would give you material support of any kind? By material support, we mean gifts, financial support for rent/school fees or cash or anything else**	- Yes<br>- No<br>- Don’t want to say<br>- Not applicable, I have never had sex
**In the last 12 months, have you provided someone (including sex workers but not necessarily only sex workers) with money, or help to pay for their expenses, or do them a favour mainly to enter into sexual relations / have sex with them, or to keep having sex with them?**	- Yes<br>- No<br>- Don’t want to say<br>- Not applicable, I have never had sex
**Thinking about the last 12 months, how often have you used condoms when you had (vaginal or anal) sex?**	- Always<br>- Most of the times<br>- Sometimes (about half the time)<br>- Rarely

	- Never<br>- Don't want to say<br>- Did not have sex in the last 12 months
**Did you use a condom the LAST time you had sex?**	- Yes<br>- No<br>- Don't want to say<br>- I have never had sex
**Have you ever been treated for a sexually transmitted infection (STI). This includes discharge from your penis or vagina, warts, sores on private parts and infections such as chlamydia, gonorrhoea, syphilis, herpes. Excludes HIV). If multiple treatments, respond for when last treated for an STI**	- Yes, in the last year<br>- Yes, more than a year ago<br>- No<br>- Don't want to say
**Have you ever been tested for STIs excluding HIV? This means having had a test for an STI such as gonorrhoea, chlamydia, herpes, HPV, syphilis, trichomonas vaginalis whether you had symptoms or not?**	- Yes, at CHIEDZA<br>- Yes, elsewhere [Specify where]_________________________<br>- No<br>- Don't want to say
**Do you currently have any of the following symptoms? (Yes/no/don't want to say)**	- Burning, stinging, pain when you pass urine (Yes/no/don't want to say)<br>- Pus or abnormal discharge coming out of vagina or penis (Yes/no/don't want to say)<br>- Abnormal smell coming out of vagina (Females only) (Yes/no/don't want to say)<br>- Itching in the genital area (Yes/no/don't want to say)<br>- Sore or blister on genitals (Yes/no/don't want to say)<br>- Bump, wart or other growth on genitals (Yes/no/don't want to say)
**Have you ever heard of drugs that can be taken to prevent HIV infection AFTER possible exposure to the virus? (Post-exposure prophylaxis or PEP)**<br>*When someone is exposed to HIV, e.g. the condom breaks or they have unprotected sex or a needlestick injury, a course of ART or ARV pills for one month can prevent HIV. This is called PEP.*	- Yes<br>- No
**Have you ever taken PEP?**	- Yes<br>- No<br>- Don't want to say<br>- I have heard about PEP but haven't taken it because I am HIV-positive<br>- I have never heard about PEP until today
**Have you ever heard of drugs that can be taken to prevent HIV infection BEFORE possible exposure to the virus? (Pre-exposure prophylaxis or PREP)**<br>*When someone does not have HIV taking a pill on an ongoing basis to prevent them from getting HIV. This is called PREP. Most people who use PREP take it everyday. It needs to be taken before sex for it to work.*	- Yes<br>- No
**Have you ever been offered PREP by a clinic or any other organisation?**	- Yes<br>- No<br>- Don't want to say<br>- I have never heard about PREP until today
**Have you ever taken PREP?**	- Yes<br>- No

	- Don't want to say<br>- I have never heard about PREP until today
**Have you been circumcised?**	- Yes<br>- No<br>- Don't want to say
**Mental Health**	
**Have you ever tried to harm or injure yourself (e.g. cutting, skin carving, extreme scratching, or burning oneself as well as punching or hitting walls to induce pain) because you were really distressed or in pain?**	- Yes<br>- No<br>- Don't want to say
**Have you ever attempted to end your life?**	- Yes<br>- No<br>- Don't want to say
**During the past 12 months have you seriously considered attempting suicide?**	- Yes<br>- No<br>- Don't want to say
**Substance use**	
**Have you ever consumed any alcohol such as beer, wine, spirits?**	- Yes<br>- No<br>- Don't want to say
**Over the last 12 months, how often have you had a drink containing alcohol?**	- 4 or more times a week<br>- 2-3 times a week<br>- 2-4 times a month<br>- Monthly or less<br>- Never<br>- Don't want to say
**In the past 12 months, how many drinks containing alcohol on average do you have on a typical day when you are drinking?**	- 1-2 drinks<br>- 3-4 drinks<br>- 5-6 drinks<br>- 7-9 drinks<br>- 10 or more<br>- Don't want to say
**In the past 12 months, how often did you have six or more drinks on one occasion?**	- Daily or almost daily<br>- Weekly (3)<br>- Monthly (2)<br>- Less than monthly (1)<br>- Never (0)<br>- Don't want to say<br>- Not applicable, I don't drink alcohol
**Do you take any of these types of drugs for non-medical reasons -i.e. for recreation/ relaxing/ forgetting?**	- Drugs you smoke (e.g. marijuana, weed, dagga, ganja) (Yes/no/don't want to say)<br>- Prescription drugs (e.g. cough solution) (Yes/no/don't want to say)

	- Drugs you sniff or inhale (e.g. glue, cocaine) (Yes/no/don't want to say)<br>- Drugs you swallow (eg. Ganja cake or popcorn) (Yes/no/don't want to say)<br>- Drugs that you inject (excluding medical drugs like insulin) (Yes/no/don't want to say)
**How often do you use drugs for non-medical reasons -i.e. for recreation/relaxing/forgetting?**	- Daily<br>- Several times a week but not daily<br>- 1-2 times a month<br>- Every few months<br>- Once or twice a year<br>- Don't want to say<br>- Not applicable, I don't use recreational drugs
**Access to digital technology**	
**Which of the following best describes your situation regarding mobile/cell phones? (select one)**	- I have my own cell phone<br>- I share the cell phone of a family member<br>- I share someone else's cell phone (not a family member)<br>- I do not use a cell phone

Supplementary table B: Univariable and multivariable associations between sociodemographic and economic factors and presence of an STI, among young people in Zimbabwe

Variable	STI prevalence N (%)	Unadjusted OR (95% CI) p-value		Age/sex/trial arm-adjusted OR (95% CI) p-value		Level 1-adjusted OR[A] (95% CI) p-value		Final Level 1 model OR[B] (95% CI) p-value	
**A priori factors**									
**Age (y)**			p<0.0001		p<0.0001		p=0.0001		p=0.0001
18 – 20	498/2885 (17.3%)	1.00		1.00		1.00		1.00	
21 – 24	609/2716 (22.4%)	1.45 (1.25 – 1.67)		1.40 (1.21 – 1.63)		1.37 (1.17 – 1.61)		1.37 (1.17 – 1.61)	
**Sex**			p<0.0001		p<0.0001		p<0.0001		p<0.0001
Male	260/2101 (12.4%)	1.00		1.00		1.00		1.00	
Female	847/2653 (24.2%)	2.25 (1.89 – 2.67)		2.22 (1.87 – 2.65)		2.11 (1.76 – 2.54)		2.11 (1.76 – 2.53)	
**Trial arm**			p=0.38		p=0.16		p=0.17		p=0.21
Control	569/2845 (20.0%)	1.00		1.00		1.00		1.00	
Intervention	538/2756 (19.5%)	0.93 (0.80 – 1.09)		0.89 (0.76 – 1.05)		0.90 (0.77 – 1.05)		0.90 (0.77 – 1.06)	
**Sociodemographic and economic factors (level 1)**									
**Province**			p=0.36		p=0.046		p=0.0086		p=0.012
Harare	524/2710 (19.3%)	1.00		1.00		1.00		1.00	
Bulawayo	583/2891 (20.2%)	1.07 (0.92 – 1.25)		1.17 (1.00 – 1.36)		1.24 (1.06 – 1.45)		1.23 (1.05 – 1.44)	
**Time lived at current address**			p=0.0001		p=0.028		p=0.098		p=0.073
More than two years	686/3800 (18.1%)	1.00		1.00		1.00		1.00	
Less than two years	421/1801 (23.4%)	1.38 (1.18 – 1.61)		1.19 (1.02 – 1.40)		1.15 (0.97 – 1.35)		1.16 (0.99 – 1.37)	
**Household wealth (N = 5584)**			p=0.0046		p=0.076		p=0.43		-
5 (richest)	210/1146 (18.3%)	1.00		1.00		1.00		-	
4	220/1220 (18.0%)	1.02 (0.80 – 1.30)		0.98 (0.77 – 1.25)		0.95 (0.74 – 1.21)		-	
3	224/1211 (18.5%)	1.10 (0.87 – 1.38)		1.04 (0.82 – 1.31)		0.99 (0.78 – 1.26)		-	
2	228/1112 (20.5%)	1.18 (0.93 – 1.49)		1.07 (0.84 – 1.35)		0.99 (0.78 – 1.26)		-	

1 (poorest)	223/903 (24.7%)	1.53 (1.19 – 1.97)		1.35 (1.05 – 1.74)		1.20 (0.91 – 1.57)		-	
**Highest completed education level**			p=0.0002		p=0.0011		p=0.092		p=0.042
Completed primary or less	262/1061 (24.7%)	1.00		1.00		1.00		1.00	
Secondary	775/4120 (18.8%)	0.69 (0.58 – 0.83)		0.74 (0.61 – 0.88)		0.81 (0.67 – 0.98)		0.79 (0.65 – 0.95)	
Post-secondary	70/420 (16.7%)	0.65 (0.48 – 0.89)		0.62 (0.45 – 0.85)		0.80 (0.56 – 1.16)		0.77 (0.54 – 1.09)	
**Current employment status**			p<0.0001		p<0.0001		p=0.0016		p=0.0010
In education or formal employment	267/1771 (15.1%)	1.00		1.00		1.00		1.00	
Informal or no employment	840/3830 (21.9%)	1.60 (1.37 – 1.88)		1.42 (1.21 – 1.67)		1.33 (1.12 – 1.59)		1.35 (1.13 – 1.61)	
**Marital status**			p<0.0001		p=0.039		p=0.069		p=0.081
Never married	765/4280 (17.9%)	1.00		1.00		1.00		1.00	
Married or living together as if married	268/1084 (24.7%)	1.49 (1.26 – 1.77)		1.04 (0.87 – 1.25)		0.91 (0.75 – 1.11)		0.94 (0.77 – 1.13)	
Divorced, widowed, or separated (and currently unmarried)	74/237 (31.2%)	2.16 (1.60 – 2.93)		1.50 (1.10 – 2.04)		1.35 (0.98 – 1.87)		1.37 (0.98 – 1.90)	
**Mobile phone ownership status (N = 5596)**			p<0.0001		p=0.50		-		-
Owns own mobile phone	984/4968 (19.8%)	1.00		1.00		-		-	
Shares or does not use a mobile phone	122/628 (19.4%)	1.05 (0.84 – 1.32)		1.08 (0.86 – 1.35)		-		-	

[A] Adjusted for age, sex, trial arm, province, time lived at current address, household wealth, education level, employment status, and marital status.

[B] Adjusted for age, sex, trial arm, province, time lived at current address, education level, employment status, and marital status

Supplementary table C: Univariable and multivariable associations between sexual behaviour and other vulnerability factors and presence of an STI, among young people in Zimbabwe

Variable	STI prevalence N (%)	Unadjusted OR (95% CI) p-value		Age/sex/trial arm-adjusted OR (95% CI) p-value		Level 1-adjusted OR[C] (95% CI) p-value		Level 1+2-adjusted OR[D] (95% CI) p-value		Final Level 2 model[E] OR (95% CI) p-value	
**Sexual behaviour and other vulnerability factors (level 2)**											
**On oral contraceptive/ implant/ Depo-Provera (N = 3500)**			p=0.13		p=0.62		p=0.84		-		-
No	661/2776 (23.8%)	1.00		1.00		1.00		-		-	
Yes	186/724 (25.7%)	1.18 (0.95 – 1.46)		1.06 (0.85 – 1.32)		0.97 (0.76 – 1.25)		-		-	
**Sexual partners in last 12 months (N = 5530)**			p<0.0001		p<0.0001		p<0.0001		p<0.0001		p<0.0001
0	238/2016 (11.8%)	1.00		1.00		1.00		1.00		1.00	
1	647/2580 (25.1%)	2.66 (2.22 – 3.18)		2.32 (1.90 – 2.82)		2.39 (1.94 – 2.95)		2.22 (1.71 – 2.86)		2.23 (1.73 – 2.88)	
2	114/532 (21.4%)	2.14 (1.58 – 2.90)		2.57 (1.87 – 3.52)		2.51 (1.83 – 3.43)		2.37 (1.67 – 3.38)		2.39 (1.69 – 3.39)	
≥3	91/402 (22.6%)	2.40 (1.76 – 3.28)		3.36 (2.40 – 4.70)		3.28 (2.33 – 4.63)		2.98 (2.03 – 4.38)		3.05 (2.09 – 4.44)	
**Transactional sex (provide/receive) in last 12 months (N = 5266)**			p=0.13		p=0.15		p=0.18		-		-
No	1027/5188 (19.8%)	1.00		1.00		1.00		-		-	
Yes	23/78 (29.5%)	1.40 (0.90 – 2.18)		1.39 (0.89 – 2.19)		1.38 (0.87 – 2.18)		-		-	
**Condom use in last 12 months during vaginal or anal sex (N = 3505)**			p=0.0008		p=0.047		p=0.034		p=0.040		p=0.044
Most of the times	284/1357 (20.9%)	1.00		1.00		1.00		1.00		1.00	
Sometimes (about half the time)	189/699 (27.0%)	1.49 (1.17 – 1.88)		1.34 (1.05 – 1.71)		1.37 (1.08 – 1.75)		1.35 (1.06 – 1.73)		1.34 (1.07 – 1.72)	
Rarely or never	375/1449 (25.9%)	1.38 (1.13 – 1.69)		1.03 (0.83 – 1.27)		1.19 (0.94 – 1.50)		1.23 (0.97 – 1.56)		1.22 (0.96 – 1.55)	

**Males only: Been circumcised (N = 2076)**			p=0.051		p=0.096		p=0.020		p=0.0076		p=0.0068
No	129/934 (13.8%)	1.00		1.00		1.00		1.00		1.00	
Yes	128/1142 (11.2%)	0.75 (0.56 – 1.00)		0.78 (0.58 – 1.05)		0.69 (0.51 – 0.94)		0.62 (0.44 – 0.88)		0.63 (0.45 – 0.88)	
**History of attempted suicide (N = 5587)**			p<0.0001		p=0.0004		p=0.0010		p=0.019		p=0.019
No	1047/5418 (19.3%)	1.00		1.00		1.00		1.00		1.00	
Yes	56/169 (33.1%)	2.17 (1.53 – 3.07)		1.89 (1.33 – 2.70)		1.83 (1.28 – 2.62)		1.58 (1.08 – 2.32)		1.58 (1.08 – 2.32)	
**High risk alcohol consumption (N = 5593)**			p=0.56		p=0.42		p=0.52		-		-
No	1075/5415 (19.9%)	1.00		1.00		1.00		-		-	
Yes	31/178 (17.4%)	0.89 (0.59 – 1.33)		1.19 (0.77 – 1.84)		1.15 (0.75 – 1.78)		-		-	
**Recreational drug use (N = 5592)**			p=0.23		p=0.074		p=0.089		p=0.69		-
No	1023/5117 (20.0%)	1.00		1.00		1.00		1.00		-	
Yes	82/475 (17.3%)	0.85 (0.65 – 1.11)		1.29 (0.98 – 1.72)		1.28 (0.96 – 1.70)		1.06 (0.78 – 1.44)		-	

[C] Adjusted for age, sex, trial arm, province, time lived at current address, education level, employment status, and marital status

[D] Adjusted for age, sex, trial arm, province, time lived at current address, education level, employment status, marital status, number of sexual partners, condom use, male circumcision, history of attempted suicide, and recreational drug use.

[E] Adjusted for age, sex, trial arm, province, time lived at current address, education level, employment status, marital status, number of sexual partners, condom use, male circumcision, and history of attempted suicide.

Supplementary table D: Univariable and multivariable associations between proximal factors and presence of an STI, among young people in Zimbabwe

Variable	STI prevalence N (%)	Unadjusted OR (95% CI) p-value		Age/sex/trial arm-adjusted OR (95% CI) p-value		Level 1+2-adjusted OR[F] (95% CI) p-value		Level 1+2+3-adjusted (Final) OR[G] (95% CI) p-value	
**Proximal factors (level 3)**									
**Accessed CHIEDZA health services**			p=0.18		p=0.13		p=0.071		p=0.049
Yes	136/766 (17.8%)	1.00		1.00		1.00		1.00	
No	971/4835 (20.1%)	1.17 (0.93 – 1.45)		1.20 (0.95 – 1.53)		1.26 (0.98 – 1.62)		1.29 (1.00 – 1.65)	
**Females only: Current pregnancy (N = 3484)**			p=0.70		p=0.68		p=0.11		-
No	783/3253 (24.1%)	1.00		1.00		1.00		-	
Yes	55/231 (23.8%)	0.93 (0.63 – 1.37)		0.92 (0.62 – 1.37)		0.72 (0.49 – 1.08)		-	
**Females only: Most recent pregnancy planning (N = 1534)**			p=0.0070		p=0.0064		p=0.090		p=0.11
Planned pregnancy	210/803 (26.2%)	1.00		1.00		1.00		1.00	
Unplanned or would have preferred to wait	235/736 (31.9%)	1.40 (1.10 – 1.78)		1.41 (1.10 – 1.81)		1.25 (0.97 – 1.63)		1.24 (0.95 – 1.62)	
**HIV status (N = 5556)**			p<0.0001		p=0.0002		p=0.0074		p=0.018
Negative	987/5205 (19.0%)	1.00		1.00		1.00		1.00	
Positive	111/351 (31.6%)	2.00 (1.52 – 2.62)		1.68 (1.28 – 2.22)		1.51 (1.12 – 2.04)		1.44 (1.07 – 1.94)	
**Ever been treated for an STI (N = 3807)**			p=0.34		p=0.40		p=0.12		-

No	822/3470 (23.7%)	1.00		1.00		1.00		-	
Yes, in the last year	50/223 (22.4%)	0.89 (0.64 – 1.25)		0.87 (0.62 – 1.22)		0.76 (0.52 – 1.09)		-	
Yes, more than a year ago	31/114 (27.2%)	1.34 (0.85 – 2.12)		1.28 (0.79 – 2.07)		1.37 (0.83 – 2.27)			
**Presence of any current symptoms (N = 5592)**			p<0.0001		p<0.0001		p=0.0023		p=0.0057
No	982/5191 (18.9%)	1.00		1.00		1.00		1.00	
Yes	124/401 (30.9%)	1.97 (1.55 – 2.50)		1.70 (1.33 – 2.16)		1.48 (1.15 – 1.90)		1.43 (1.11 – 1.84)	
**Taken PEP (N = 5586)**			p=0.37		p=0.49		p=0.92		-
No	1093/5535 (19.8%)	1.00		1.00		1.00		-	
Yes	13/48 (27.1%)	1.37 (0.69 – 2.70)		1.29 (0.62 – 2.67)		0.96 (0.43 – 2.15)		-	
**Been offered PrEP (N = 5585)**			p<0.0001		p=0.0007		p=0.032		p=0.025
No	1066/5468 (19.5%)	1.00		1.00		1.00		1.00	
Yes	40/116 (34.5%)	2.24 (1.53 – 3.28)		1.99 (1.33 – 2.96)		1.57 (1.04 – 2.38)		1.61 (1.06 – 2.43)	
**Taken PrEP (N = 5585)**			p=0.30		p=0.61		p=0.59		-
No	1090/5520 (19.8%)	1.00		1.00		1.00		-	
Yes	16/65 (24.6%)	1.35 (0.77 – 2.38)		1.17 (0.64 – 2.15)		0.85 (0.47 – 1.54)		-	

[F] Adjusted for age, sex, trial arm, province, time lived at current address, education level, employment status, marital status, number of sexual partners, condom use, male circumcision, and history of attempted suicide.

[G] Adjusted for age, sex, trial arm, province, time lived at current address, education level, employment status, marital status, number of sexual partners, condom use, male circumcision, history of attempted suicide, CHIEDZA service access, pregnancy planning, HIV status, current symptoms, and if been offered PrEP.

Supplementary table E: Univariable, age- and trial arm-adjusted, and multivariable associations between variables and presence of an STI, among female youth in Zimbabwe

Variable	STI prevalence N (%)	Unadjusted OR (95% CI) p-value		Age/trial arm-adjusted OR (95% CI) p-value		Final Level 1 model OR[B] (95% CI) p-value	
**A priori factors**							
**Age (y)**			p=0.0001		p=0.0001		p=0.0054
18 – 20	380/1735 (21.9%)	1.00		1.00		1.00	
21 – 24	467/1765 (26.5%)	1.38 (1.17 – 1.62)		1.38 (1.17 – 1.62)		1.29 (1.08 – 1.53)	
**Trial arm**			p=0.14		p=0.12		p=0.13
Control	425/1697 (25.0%)	1.00		1.00		1.00	
Intervention	422/1803 (23.4%)	0.87 (0.73 – 1.05)		0.87 (0.72 – 1.04)		0.87 (0.72 – 1.04)	
**Sociodemographic and economic factors (level 1)[A]**							
**Current employment status**			p<0.0001		p<0.0001		p<0.0001
In education or formal employment	174/941 (18.5%)	1.00		1.00		1.00	
Informal or no employment	673/2559 (26.3%)	1.67 (1.38 – 2.02)		1.59 (1.31 – 1.93)		1.60 (1.30 – 1.95)	
**Marital status**			p=0.0077		p=0.078		p=0.12
Never married	527/2291 (23.0%)	1.00		1.00		1.00	
Married or living together as if married	252/993 (25.4%)	1.17 (0.97 – 1.40)		1.06 (0.87 – 1.28)		0.94 (0.76 – 1.15)	
Divorced, widowed, or separated (and currently unmarried)	68/216 (31.5%)	1.63 (1.18 – 2.25)		1.46 (1.05 – 2.03)		1.33 (0.95 – 1.85)	
**Sexual behaviour and other vulnerability factors (level 2)[B]**							
**Sexual partners in last 12 months (N = 3472)**			p< 0.0001		p<0.0001		p< 0.0001
0	175/1193 (14.7%)	1.00		1.00		1.00	
1	562/1993 (28.2%)	2.49 (2.03 – 3.07)		2.47 (1.98 – 3.09)		2.32 (1.71 – 3.15)	

2	67/202 (33.2%)	3.00 (2.11 – 4.26)		2.94 (2.06 – 4.20)		2.53 (1.72 – 3.72)	
≥3	34/84 (40.5%)	4.18 (2.49 – 7.02)		4.13 (2.43 – 7.01)		3.41 (1.94 – 5.96)	
**Condom use in last 12 months during vaginal or anal sex (N = 2272)**			p= 0.051		p=0.069		p=0.048
Most of the times	180/624 (28.9%)	1.00		1.00		1.00	
Sometimes (about half the time)	141/412 (34.2%)	1.39 (1.04 – 1.85)		1.37 (1.03 – 1.83)		1.44 (1.08 – 1.93)	
Rarely or never	338/1236 (27.4%)	1.02 (0.80 – 1.30)		1.02 (0.80 – 1.30)		1.23 (0.93 – 1.64)	
**History of attempted suicide (N = 3491)**			p= 0.0055		p=0.0049		p=0.050
No	796/3354 (23.7%)	1.00		1.00		1.00	
Yes	48/137 (35.0%)	1.70 (1.17 – 2.46)		1.71 (1.18 – 2.48)		1.49 (1.00 – 2.21)	
**Proximal factors (level 3)[C]**							
**Accessed CHIEDZA health services**			P=0.051		p=0.17		p=0.034
Yes	111/546 (20.3%)	1.00		1.00		1.00	
No	736/2954 (24.9%)	1.28 (1.00 – 1.64)		1.20 (0.92 – 1.57)		1.34 (1.02 – 1.75)	
**Females only: Most recent pregnancy planning (N = 1539)**			p=0.0070		p=0.0064		p=0.10
Planned pregnancy	210/803 (26.2%)	1.00		1.00		1.00	
Unplanned or would have preferred to wait	235/736 (31.9%)	1.40 (1.10 – 1.78)		1.41 (1.10 – 1.81)		1.25 (0.96 – 1.63)	
**HIV status (N = 3474)**			p=0.0001		p=0.0005		p=0.031
Negative	744/3202 (23.2%)	1.00		1.00		1.00	
Positive	96/272 (35.3%)	1.82 (1.34 – 2.47)		1.72 (1.27 – 2.33)		1.44 (1.03 – 2.01)	
**Presence of any current symptoms (N = 3496)**			p=0.0002		p=0.0006		p=0.0046
No	742/3181 (23.3%)	1.00		1.00		1.00	
Yes	104/315 (33.0%)	1.67 (1.27 – 2.19)		1.62 (1.23 – 2.13)		1.33 (1.00 – 1.77)	

**Been offered PrEP (N = 3493)**			p=0.0006		p=0.0016		p=0.022
No	814/3410 (23.9%)	1.00		1.00		1.00	
Yes	33/83 (39.8%)	2.18 (1.40 – 3.39)		2.10 (1.33 – 3.32)		1.72 (1.08 – 2.74)	

[A] Adjusted for age, trial arm, employment status, and marital status

[B] Adjusted for age, trial arm, employment status, marital status, number of sexual partners, condom use, and history of attempted suicide

[C] Adjusted for age, trial arm, employment status, marital status, number of sexual partners, condom use, history of attempted suicide, CHIEDZA service access, pregnancy planning, HIV status, current symptoms, and if been offered PrEP.

Supplementary table F: Univariable, age- and trial arm-adjusted, and multivariable associations between variables and presence of an STI, among male youth in Zimbabwe

Variable	STI prevalence N (%)	Unadjusted OR (95% CI) p-value		Age/sex/trial arm-adjusted OR (95% CI) p-value		Final Level 1 model OR[B] (95% CI) p-value	
**A priori factors**							
**Age (y)**			p=0.012		p=0.012		p=0.0071
18 – 20	118/1150 (10.3%)	1.00		1.00		1.00	
21 – 24	142/951 (14.9%)	1.47 (1.09 – 1.98)		1.47 (1.09 – 1.98)		1.51 (1.12 – 2.04)	
**Trial arm**			p=0.86		p=0.82		p=0.78
Control	144/1148 (12.5%)	1.00		1.00		1.00	
Intervention	116/953 (12.2%)	0.97 (0.72 – 1.32)		0.97 (0.71 – 1.31)		0.96 (0.71 – 1.29)	
**Sociodemographic and economic factors (level 1)[A]**							
**Province**			p=0.038		p=0.018		p=0.018
Harare	96/900 (10.7%)	1.00		1.00		1.00	
Bulawayo	164/1201 (13.7%)	1.37 (1.02 – 1.85)		1.43 (1.06 – 1.93)		1.43 (1.06 – 1.93)	
**Sexual behaviour and other vulnerability factors (level 2)[B]**							
**Sexual partners in last 12 months (N = 2058)**			p <0.0001		p= 0.0003		p= 0.0003
0	63/823 (7.7%)	1.00		1.00		1.00	
1	85/587 (14.5%)	2.06 (1.42 – 2.98)		1.97 (1.33 – 2.91)		1.96 (1.32 – 2.91)	
2	47/330 (14.2%)	2.13 (1.26 – 3.62)		2.07 (1.19 – 3.59)		2.10 (1.20 – 3.66)	
≥3	57/318 (17.9%)	2.87 (1.83 – 3.51)		2.71 (1.69 – 4.35)		2.79 (1.74 – 4.49)	
**Males only: Been circumcised (N = 2076)**			p=0.051		p=0.096		p= 0.0099
No	129/934 (13.8%)	1.00		1.00		1.00	
Yes	128/1142 (11.2%)	0.75		0.78 (0.58 – 1.05)		0.65	

		(0.56 – 1.00)				(0.47 – 0.90)	
**History of attempted suicide (N = 2096)**			p= 0.0054		p= 0.0047		p=0.041
No	251/2064 (12.2%)	1.00		1.00		1.00	
Yes	8/32 (25.0%)	3.23 (1.42 – 7.39)		3.21 (1.43 – 7.20)		2.74 (1.04 – 7.18)	
**Proximal factors (level 3)[C]**							
**Presence of any current symptoms (N = 2096)**			p=0.010		P=0.015		p=0.036
No	240/2010 (11.9%)	1.00		1.00		1.00	
Yes	20/86 (23.3%)	2.17 (1.20 – 3.92)		2.09 (1.16 – 3.78)		1.89 (1.04 – 3.43)	

[A] Adjusted for age, trial arm, and province
[B] Adjusted for age, trial arm, province, number of sexual partners, and history of attempted suicide
[C] Adjusted for age, trial arm, province, number of sexual partners, history of attempted suicide and presence of current symptoms

Supplementary table G: Univariable associations between sociodemographic and economic factors and whether an individual has accessed CHIEDZA services within the trial arm (N = 2756 unless otherwise stated)

**Variable**	**Accessed CHIEDZA N (%)**	**OR (95% CI) p-value**	
**Age (y)**			p=0.60
18 – 20	401/1402 (28.6%)	1.00	
21 – 24	348/1354 (25.7%)	0.96 (0.81 – 1.13)	
**Sex**			p=0.0002
Male	219/953 (23.0%)	1.00	
Female	530/1803 (29.4%)	1.52 (1.22 – 1.90)	
**Province**			p=0.68
Harare	357/1376 (25.9%)	1.00	
Bulawayo	392/1380 (28.4%)	0.94 (0.70 – 1.27)	
**Time lived at current address**			P<0.0001
More than two years	599/1813 (33.0%)	1.00	
Less than two years	150/943 (15.9%)	0.53 (0.42 – 0.66)	
**Household wealth (N = 2749)**			p=0.60
5 (richest)	142/564 (25.2%)	1.00	
4	157/578 (27.2%)	1.03 (0.78 – 1.38)	
3	173/559 (31.0%)	1.17 (0.89 – 1.52)	
2	154/525 (29.3%)	1.16 (0.86 – 1.56)	
1 (poorest)	120/523 (22.9%)	0.99 (0.71 – 1.38)	
**Highest completed education level**			p=0.0082
Completed primary or less	125/541 (23.1%)	1.00	
Secondary	580/1997 (29.0%)	1.42 (1.11 – 1.81)	
Post-secondary	44/218 (20.2%)	1.08 (0.72 – 1.63)	

**Current employment status**			p=0.83
In education or formal employment	232/842 (27.6%)	1.00	
Informal or no employment	517/1914 (27.0%)	1.02 (0.84 – 1.25)	
**Marital status**			p=0.83
Never married	559/2061 (27.1%)	1.00	
Married or living together as if married	160/592 (27.0%)	1.08 (0.84 – 1.39)	
Divorced, widowed, or separated (and currently unmarried)	30/103 (29.1%)	1.02 (0.69 – 1.52)	
**Mobile phone ownership status (N = 2754)**			p=0.63
Owns own mobile phone	655/2433 (26.9%)	1.00	
Shares or does not use a mobile phone	93/321 (29.0%)	1.06 (0.83 – 1.35)	
